# Supplementary material for: Causal Relationship between Aging and Anorexia Nervosa: A White-Matter-Microstructure-Mediated Mendelian Randomization Analysis
Source: Biomedicines. 2024 Aug 16;12(8):1874. doi: 10.3390/biomedicines12081874 (PMC11351342; doi:10.3390/biomedicines12081874)
Supplement: Supplementary file 1 [file biomedicines-12-01874-s001.zip › Supplementary Figure/Figure S7/Single SNP plot of MR analysis for ubm-b-1960 to AN.pdf]

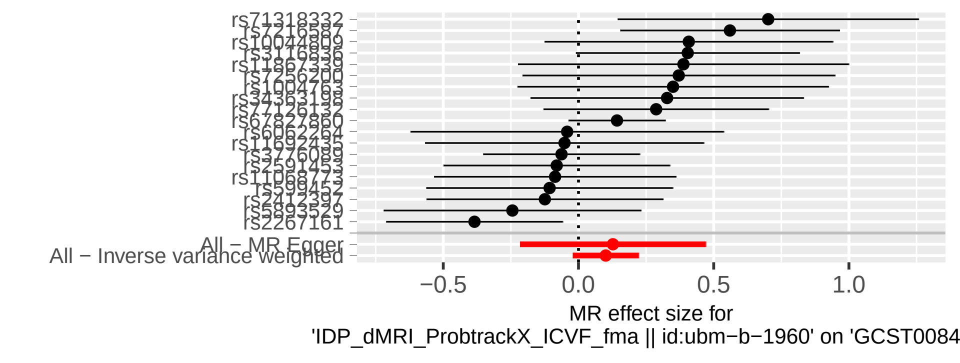
rs71318332
rs72165587
rs10044809
rs3116836
rs11867339
rs7256200
rs1004763
rs34363198
rs77126132
rs67827860
rs6062264
rs11692435
rs3776089
rs2591453
rs11068773
rs599452
rs2412397
rs5893529
rs2267161
All − MR Egger
All − Inverse variance weighted
−0.5
0.0
0.5
1.0
MR effect size for
'IDP_dMRI_ProbtrackX_ICVF_fma || id:ubm−b−1960' on 'GCST0084
